# To Stick or Not to Stick: The Multiple Roles of Cell Adhesion Molecules in Neural Circuit Assembly

**DOI:** 10.3389/fnins.2022.889155

**Published:** 2022-04-28

**Authors:** Trevor Moreland, Fabienne E. Poulain

**Affiliations:** Department of Biological Sciences, University of South Carolina, Columbia, SC, United States

**Keywords:** growth cone, axon targeting, pathfinding, synaptic specificity, signaling, contact

## Abstract

Precise wiring of neural circuits is essential for brain connectivity and function. During development, axons respond to diverse cues present in the extracellular matrix or at the surface of other cells to navigate to specific targets, where they establish precise connections with post-synaptic partners. Cell adhesion molecules (CAMs) represent a large group of structurally diverse proteins well known to mediate adhesion for neural circuit assembly. Through their adhesive properties, CAMs act as major regulators of axon navigation, fasciculation, and synapse formation. While the adhesive functions of CAMs have been known for decades, more recent studies have unraveled essential, non-adhesive functions as well. CAMs notably act as guidance cues and modulate guidance signaling pathways for axon pathfinding, initiate contact-mediated repulsion for spatial organization of axonal arbors, and refine neuronal projections during circuit maturation. In this review, we summarize the classical adhesive functions of CAMs in axonal development and further discuss the increasing number of other non-adhesive functions CAMs play in neural circuit assembly.

## Introduction

Forming precise neural circuits is critical for nervous system function. Defects in neuronal connectivity have notably been observed in multiple neurodevelopmental disorders including fragile X syndrome (Swanson et al., 2018), autism spectrum disorders (McFadden and Minshew, 2013; Di Martino et al., 2014; Avital et al., 2015), tuberous sclerosis complex (Widjaja et al., 2010; Baumer et al., 2015; Im et al., 2016) and others, making the wiring of axonal connections a subject of intense research.

Developing axons navigate along precise paths toward their target by responding to attractive and repulsive guidance cues present in their environment. Navigation is ensured by highly motile structures at the leading end of axons, the growth cones, which harbor a unique repertoire of receptors at their surface that allow them to interpret the various extracellular signals they encounter. Many secreted and membrane-anchored factors including cell adhesion molecules (CAMs) (Tessier-Lavigne and Goodman, 1996), the canonical guidance cues Ephrins, Netrins, Semaphorins, and Slits (Dickson, 2002), neurotrophic and growth factors (Charoy et al., 2012), and morphogens (Sánchez-Camacho and Bovolenta, 2009; Yam and Charron, 2013), provide long-range or contact-mediated signals. Growth cones integrate the guidance information they receive from these signals and in turn, transduce the mechanical forces required for axon extension and turning (Kerstein et al., 2015). After reaching their final destination, axons stop elongating, branch extensively to form a terminal arbor and establish specific synaptic connections with appropriate partners. Patterns of connectivity are subsequently remodeled and refined in an activity-dependent manner, leading to the establishment of precise local circuits for an efficient transfer of information (Kutsarova et al., 2017). Axon pathfinding, selective target innervation and specificity of synapse formation are central aspects of circuit wiring that all critically rely on long-range as well as contact-mediated signaling between axons and their substrate or surrounding cells.

**FIGURE 1:**
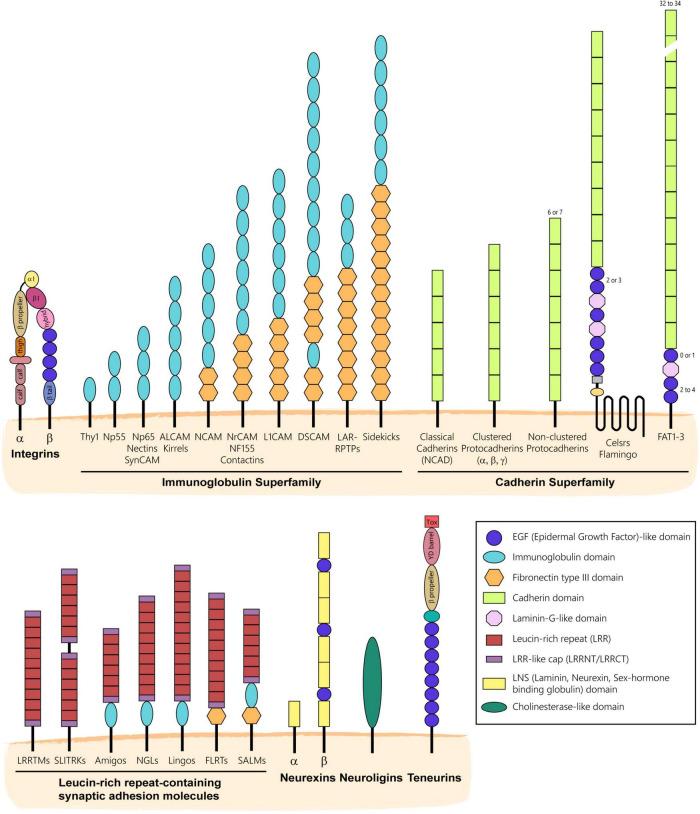
Main families of CAMs expressed in the nervous system. CAMs have historically been classified into families based on the structural composition of their extracellular domain. Integrins form obligate heterodimers composed of α and β integrin subunits that cluster at the plasma membrane to mediate adhesion to the extracellular matrix. CAMs of the Immunoglobulin Superfamily (IgSF) are characterized by the presence of one or more Ig-like domains that can be followed by Fibronectin type III domain (Fn3) repeats. IgSF CAMs mediate adhesion by engaging in homophilic or heterophilic interactions. Most of them include a transmembrane and intracellular domains, but some like Contactins are GPI-anchored. CAMs of the Cadherin Superfamily mostly engage in homophilic interactions and are characterized by the presence of one or more calcium-binding cadherin repeats. The Leucine-rich repeat (LRR) Superfamily includes adhesion molecules that are characterized by the presence or LRRs and can include Ig or Fn3 domains in their extracellular domain. LRR CAMs are often found at synapses and engage in both homophilic or heterophilic *trans*-interactions. Neurexins and Neuroligins engage in heterophilic interactions in *trans* at nascent synapses to promote synaptic differentiation and stabilization. Teneurins are type II single-pass transmembrane proteins that interact homophilically or engage in *trans*-interactions with Latrophilins, a class of adhesion G-protein coupled receptors (not shown).

CAMs form a very large group of transmembrane or glycophosphatidylinositol (GPI)-anchored proteins that mediate contacts between cells or cells and a substrate *via* homophilic or heterophilic interactions. First identified in the mid-70s as molecules mediating cell-cell adhesion (Rutishauser et al., 1976), CAMs have historically been classified into four main families including integrins, CAMs of the immunoglobulin superfamily (IgSF), cadherins, and selectins based on the structural composition of their extracellular domain (Bock, 1991). However, this classification omits molecules discovered afterward that also act as cell adhesion proteins such as neuroligins, neurexins, teneurins, and synaptic proteins containing extracellular leucine-rich repeats (LRRs) (Figure 1). Thanks to their diverse extracellular domains conferring distinct adhesion modalities and engaging in unique protein interactions, CAMs regulate many aspects of neural development ranging from neurogenesis (Homan et al., 2018; Huang et al., 2020) and neuronal migration (Schmid and Maness, 2008; Solecki, 2012; Chen et al., 2018) to neurite development (Pollerberg et al., 2013; Missaire and Hindges, 2015), synaptogenesis (Gerrow and El-Husseini, 2006; Yogev and Shen, 2014) and myelination (Rasband and Peles, 2021). Not surprisingly, many mutations in CAMs have been linked to neurodevelopmental and psychiatric disorders (Sakurai, 2017; Cuttler et al., 2021; Jaudon et al., 2021). Interestingly, whereas CAMs have long been known to regulate axonal development and synaptogenesis through their adhesive properties, other non-adhesive and perhaps counterintuitive functions of CAMs have more recently emerged. In this review, we summarize the classical adhesive functions of CAMs in axonal and synaptic development and further discuss the increasing number of other non-adhesive roles CAMs play in neural circuit assembly and maturation.

## Adhesive Functions of CAMs in Circuit Wiring

Through their adhesive properties, CAMs mediate stabilizing contacts and attachment between axons and their surrounding environment that are critical for axon navigation, fasciculation, target selection, and synaptogenesis.

### Interactions With the Extracellular Matrix and Glial Cells for Axon Navigation

**FIGURE 2:**
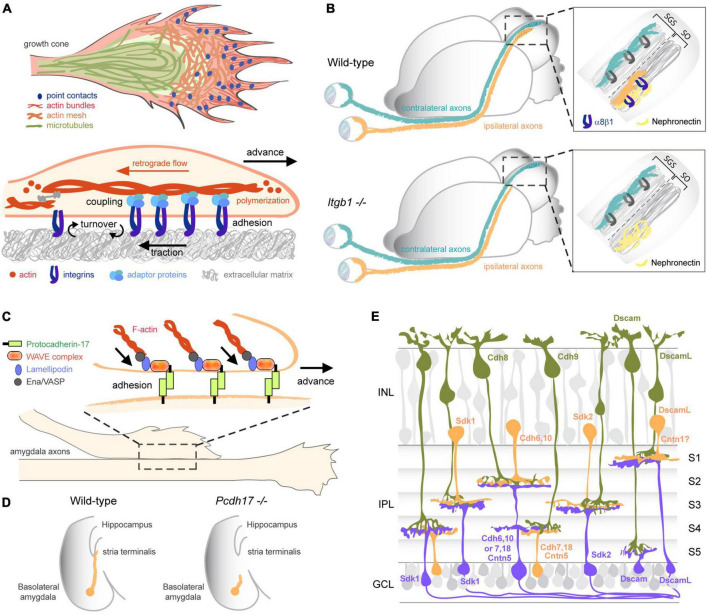
Adhesive functions of CAMs in circuit wiring. **(A)** Integrins mediate adhesion to the ECM for growth cone advance. Binding of integrins to ECM ligands leads to the clustering of integrins and the recruitment of adaptor proteins linking integrins to the actin cytoskeleton. The point contacts hence formed promote growth cone advance by stabilizing filopodial protrusions and restraining the retrograde flow of actin at the growth cone periphery. **(B)** Integrins mediate adhesion to the ECM for target selection. In the visual system, integrin α8β1 is selectively expressed by retinal ganglion cells projecting ipsilaterally. Its ligand, the ECM glycoprotein Nephronectin, is restricted to a sublamina at the target. Interaction between integrin α8β1 and Nephronectin is necessary for the laminar targeting of ipsilateral axons to the rostral stratum opticum (SO). Deleting integrin α8β1 or Nephronectin causes a dramatic loss of ipsilateral projections while contralateral projections remain unaffected in the stratum griseum superficial (SGS). Adapted from Su et al. (2021). **(C,D)** Protocadherin-17 (Pcdh17) mediates *trans*-axonal interactions for proper tract formation. **(C)** Pcdh17 accumulates at homotypic contacts between growth cones and axons from amygdala neurons, where it recruits the WAVE complex, Lamellipodin, and Ena/VASP proteins that remodel the actin cytoskeleton and promote membrane protrusion. Pcdh17-mediated adhesion enhances growth cone motility and enable growth cone advance along homotypic axons. **(D)** Pcdh17 is required for the extension of amygdala axons through the stria terminalis toward the hypothalamus. Adapted from Hayashi et al. (2014). **(E)** A CAM code specifies laminar targeting and synaptic specificity in the retina. Sdk1, Sdk2, Dscam, DscamL, and Cntns (not all a represented here) are expressed in non-overlapping subsets of bipolar (green), amacrine (orange), and retinal ganglion cells (purple) and engage in homophilic *trans-*interactions to direct synapse formation between matching partners in specific laminae (S1–S5) of the inner plexiform layer (IPL). Classical cadherins (Cdh) also contribute to the molecular code specifying connections. INL, inner nuclear layer; GCL, ganglion cell layer. Adapted from Sanes and Zipursky (2020).

Proper axon pathfinding requires a tightly controlled adhesion of growth cones to their substrate. The assembly and detachment of adhesions to the extracellular matrix (ECM) is especially critical for the advance of pioneer axons that are the first to extend in a particular region. Like non-neuronal cells that form integrin-mediated focal adhesions at their leading edge during migration (Mishra and Manavathi, 2021), growth cones assemble similar integrin-dependent adhesions named point contacts with the ECM (Gomez et al., 1996; Woo and Gomez, 2006). Point contacts form after integrins at the surface of growth cones bind ECM ligands, leading to the clustering of integrins and subsequent recruitment of adaptor proteins linking integrins to the actin cytoskeleton (Figure 2A). By stabilizing filopodial protrusions and restraining the retrograde flow of actin at the growth cone periphery, point contacts promote the advance of the growth cone and axon extension (Woo and Gomez, 2006; Myers and Gomez, 2011; Nichol et al., 2016; Kershner and Welshhans, 2017). Interestingly, many extracellular factors regulate axon elongation and cell migration by modulating integrin-mediated adhesions (Nakamoto et al., 2004; Bechara et al., 2008). Substrates or guidance cues that promote or inhibit the localized assembly and turnover of point contacts lead to growth cone turning *in vitro* (Hines et al., 2010; Myers and Gomez, 2011; Nichol et al., 2016; Kerstein et al., 2017), further suggesting a direct role for integrin-mediated attachment in axon pathfinding. In mammals, 18 α and 8 β integrins can assemble into 24 heterodimers that bind with different affinities to a large diversity of ECM ligands (Hynes, 2002). Inhibiting integrin β1 that forms the majority of integrin heterodimers reduces retinal axon elongation in zebrafish (Lilienbaum et al., 1995). It also prevents ipsilateral retinal projections from innervating a specific sublamina of the superior colliculus that expresses the ECM glycoprotein Nephronectin in mouse (Su et al., 2021; Figure 2B). Thus, integrin-mediated interactions between growth cones and the ECM not only regulate the rate of axon elongation but also directly specify target selection for circuit wiring. To what extent a cell-specific integrin code generated by the different combinations of α and β integrins contributes to the specificity of network assembly remains to be elucidated.

In addition to adhering to the ECM, pioneer growth cones directly interact with surrounding glial cells such as neuroepithelial cells, radial glial cells, and astrocytes during their navigation (Rigby et al., 2020). Glial cells often localize at intermediate choice points along migratory routes where they act as guideposts providing contact-mediated spatial information or acting as a permissive substrate for growth. For instance, radial glia create a palisade at the optic chiasm where they directly contact and guide retinal axons (Marcus et al., 1995). More recently, neural stem cells residing in the ventricular zone of the forebrain medial ganglionic eminence have been shown to use their radial fiber scaffold to direct corticospinal axons at the junction between the striatum and globus pallidus (Kaur et al., 2020). Most adhesive contacts between navigating growth cones and glial cells appear to be mediated by CAMs of the Immunoglobulin and Cadherin superfamilies. N-cadherin and NCAM, for instance, promote retinal axon outgrowth over astrocytes *in vitro* (Neugebauer et al., 1988). They also mediate strong interactions between growth cones of olfactory axons and ensheathing cells *in vivo*, enabling olfactory axons to ride along ensheathing cell bodies as they pioneer the path toward the olfactory bulb (Su and He, 2010). In the spinal cord, NrCAM expressed by floor plate cells guides commissural axons across the midline by, in part, interacting with Contactin-2 (Cntn2, also known as TAG-1 or axonin-1) at the axonal surface (Stoeckli et al., 1997; Fitzli et al., 2000). NrCAM is also expressed by radial glia at the optic chiasm and promotes the crossing of NrCAM-positive retinal axons projecting contralaterally (Williams et al., 2006; Kuwajima et al., 2012). Along the optic tract, NF-protocadherin, a member of the Cadherin superfamily, mediates interactions between retinal axons and their neuroepithelial substrate for proper pathfinding (Leung et al., 2013). Retinal axons expressing L1CAM are then guided in the superior colliculus by collicular cells expressing ALCAM (also called BEN, DM-GRASP, SC1, or Neurolin) (Buhusi et al., 2009). In the absence of ALCAM, axonal branches fail to extend mediolaterally, leading to defects in retinotopic map formation. Outside the visual system, recent work has revealed that Celsr3, a member of the Flamingo group within the Cadherin superfamily, is present at the surface of commissural growth cones and promotes axon pathfinding across the floor plate by binding in *trans* to Dystroglycan, a transmembrane protein at the surface of neurepithelial cells (Lindenmaier et al., 2019). Interestingly, other families of CAMs such as teneurins and neuroligins have been found to accumulate in growth cones during axon elongation (Suzuki et al., 2014; Gatford et al., 2021). Determining whether they also mediate adhesion between pioneer growth cones and glial cells will be important to gain a more comprehensive view of the inter-cellular interactions that govern neural circuit assembly during early development.

### Axon Fasciculation for Tract Formation

Pioneer axons play a critical role in neural circuit assembly, not only by defining first itineraries toward appropriate targets, but also by acting as guides and providing a scaffold for later-born axons that follow them (Pike et al., 1992; Hidalgo and Brand, 1997; Rash and Richards, 2001). In the olfactory and retinotectal systems, for instance, ablation of early-born pioneer neurons causes follower axons to misroute and fail to build proper connections (Whitlock and Westerfield, 1998; Pittman et al., 2008; Okumura et al., 2016). The formation of axon tracts en route to a target involves homotypic or heterotypic fasciculation between axons, which are usually initiated after a growth cone contacts the shaft of a neighboring axon and moves along it. Alternatively, axon shafts can dynamically interact, leading to a zippering behavior triggering their fasciculation (Šmít et al., 2017). Extensive literature has demonstrated a major role for IgSF CAMs in regulating homotypic axon-axon interactions (Spead and Poulain, 2021). CAMs engaged in homophilic (between same CAMs) or heterophilic (between different CAMs) *trans*-interactions mediate the selective recognition between elongating growth cones and pre-existing axon shafts, thereby dictating the specificity of axonal bundling for tract formation. They also provide the adhesive force required for growth cone advance through their coupling to the actin cytoskeleton (Pollerberg et al., 2013; Abe et al., 2018). Not surprisingly, the loss of specific CAMs leads to disorganized tracts in many circuits. Blocking L1CAM in the chick hindlimb, for instance, causes a defasciculation of both motor and sensory axons that fail to project to their respective targets (Landmesser et al., 1988; Honig and Rutishauser, 1996; Honig et al., 2002). L1CAM is also required for the fasciculation between axons innervating the peduncle of the mushroom bodies in Drosophila (Siegenthaler et al., 2015). Likewise, ALCAM and DSCAM both regulate the fasciculation of retinal axons in the visual system (Pollerberg and Mack, 1994; Ott et al., 1998; Weiner et al., 2004; Bruce et al., 2017). Continuous synthesis of ALCAM in growth cones is notably required for the preferential growth of retinal axons on ALCAM substrates and maintained by local mRNA translation (Thelen et al., 2012). In the mouse motor system, Cntn2 accumulates in the distal segment of motor axons extending in the periphery and controls their fasciculation (Suter et al., 2020). Conversely in the peripheral system, SynCAM2 and SynCAM3 were found to regulate contacts between sensory afferents as they enter the dorsal root entry zone of the spinal cord (Frei et al., 2014). Similarly to IgSF CAMs, members of the Cadherin superfamily have also emerged as important regulators of tract organization. Tectofugal projections innervating different parts of the brain elongate along pre-existing axonal pathways expressing the same cadherin, demonstrating that cadherins mediate selective axon fasciculation through homotypic *trans*-interactions (Treubert-Zimmermann et al., 2002). As such, cadherins organize axonal tracts depending on their selective expression in the nervous system. N-cadherin and Cadherin-8, for instance, are both required for the fasciculation of mossy fibers in the hippocampus (Bekirov et al., 2008), while Cadherin-11 promotes the bundling of motor axons (Marthiens et al., 2005). More recently, Protocadherin-17 (Pcdh17) has been found to regulate the formation of homotypic contacts between amygdala axons elongating toward the hypothalamus and ventral striatum (Hayashi et al., 2014; Figures 2C,D). Growth cones lacking Pdch17 no longer migrate along Pdch17-positive axons, whereas axons ectopically expressing Pdch17 intermingle with axons expressing endogenous Pdch17.

Guidance cues can regulate axon fasciculation and pathfinding by modulating the levels of CAMs at the axonal surface. Semaphorin3D, for instance, promotes the bundling of medial longitudinal fascicle axons in zebrafish by increasing L1CAM protein levels (Wolman et al., 2007). In Drosophila, Semaphorin-1a reverse signaling promotes the fasciculation of photoreceptor axons by inhibiting Rho1, a small GTPase known to mediate the degradation of the NCAM ortholog Fasciclin 2 (Hsieh et al., 2014). Conversely in the Xenopus visual system, Semaphorin-3A (Sema3A) prevents retinal axons from exiting their normal trajectories by inducing the local translation of NF-protocadherin in retinal growth cones (Leung et al., 2013). Alternatively, extracellular factors can regulate the strength of growth cone adhesion by modulating the coupling of CAMs to the actin cytoskeleton. Netrin-1, for instance, promotes traction force for growth cone migration by enhancing the coupling of L1CAM to F-actin *via* the adaptor Shootin1a (Kubo et al., 2015; Baba et al., 2018). Whether other guidance cues such as Ephrins (Luxey et al., 2013) or Slits (Jaworski and Tessier-Lavigne, 2012) regulate axon fasciculation by modulating CAMs and their adhesive properties remains to be determined.

### *Trans-*Interactions for Synaptic Specificity

CAMs not only regulate axon growth, fasciculation and guidance toward a main target but also dictate the specificity of synapse formed between axons and dendrites. After reaching their main target area, axons must establish synapses with appropriate partners while avoiding unsuitable ones. Synaptic specificity is achieved by both laminar targeting, during which axon terminals and dendrites of post-synaptic neurons sharing similar functional properties assemble into local layers within the main target, and specific cellular and sub-cellular synapse assembly.

Studies on the mechanisms governing laminar targeting in the visual system have demonstrated critical roles for IgSF CAMs and cadherins in mediating *trans*-cellular recognition between correct synaptic partners (Huberman et al., 2010; Sanes and Zipursky, 2020). In the chick retina, Sidekick 1 (Sdk1), Sdk2, Dscam, DscamL, and Cntns were found to be uniquely expressed in non-overlapping subsets of two classes of interneurons, the bipolar and amacrine cells, as well as in their post-synaptic partners, the retinal ganglion cells (RGCs) (Figure 2E). Interestingly, RGCs and interneurons with matching expression of these IgSF CAMs form synapses in specific sublaminae of the inner plexiform layer (Yamagata et al., 2002; Yamagata and Sanes, 2008, 2012). Modifying the “CAM code” a neuron expresses by depleting or overexpressing any of these CAMs diverts axonal arbors to sublaminae expressing the matching set of CAMs, indicating an essential role for homophilic *trans*-interactions in laminar targeting. Similar laminar targeting defects were observed in mice lacking Sdks, DSCAM, or Cntn5, suggesting conserved roles for these CAMs across vertebrates (Fuerst et al., 2010; Krishnaswamy et al., 2015; Peng et al., 2017; Yamagata and Sanes, 2019). However, whereas Sdks and Cntn5 regulate laminar targeting by homophilic interactions in both mouse and chick, DSCAM appears to exert its function differently in mouse by masking signaling from cadherins (Simmons et al., 2017; Garrett et al., 2018). Indeed, cadherins also contribute significantly to the coding of laminar targeting through their homophilic interactions. Among the 15 different classical cadherins detected in the direction-selective circuits in the retina, six are expressed in combination or individually in populations of functionally distinct interneurons and RGCs and control their laminar connectivity (Duan et al., 2014, 2018). Cadherin-8 (Cdh8) and Cdh9, for instance, direct axons of a subset of bipolar cells to RGCs responding to bright or dark moving objects, whereas Cdh7 and Cdh18 specify synapses formed between amacrine cells and RGCs responding to nasal motion. Cdh6, on the other hand, targets not only amacrine cell axons to RGCs responding to ventral motion in the retina, but also RGC axons to their specific visual targets in the brain (Osterhout et al., 2011). A similar coding principle for axon-target matching has been observed in other circuits. In the hippocampus, for instance, Cdh9 specifically regulates the formation of mossy fiber synapses between dentate gyrus and CA3 neurons (Williams et al., 2011). Likewise in the cerebellum, Cdh7 mediates synapse formation between pontine axons and granule neurons (Kuwako et al., 2014). More recently, IgSF11 has been identified as the homophilic adhesion molecule controlling synapse formation between inhibitory chandelier cells and pyramidal neurons in the cortex (Hayano et al., 2021).

Other CAMs besides IgSF CAMs and cadherins regulate synaptic specificity. Teneurins, for instance, are expressed in several inter-connected regions in the nervous system and form *trans*-synaptic interactions by binding homophilically or heterophilically to Latrophilins, a class of adhesion G-protein coupled receptors (Boucard et al., 2014; Araç and Li, 2019). The role of teneurins in synaptic partner matching was first established in Drosophila, in which Ten-a and Ten-m instruct target selection in the motor and olfactory systems (Hong et al., 2012; Mosca et al., 2012). Teneurins were found afterward to also specify connectivity in the hippocampus and the visual system of vertebrates. Teneurin-2 (Tenm2) promotes synapse formation between CA3 Schaffer collaterals and CA1 pyramidal hippocampal neurons by forming a *trans*-synaptic complex with Latrophilin-3 (Lphn3) and FLRT3 (Sando et al., 2019). In contrast, Tenm3 homophilic interactions are required for the precise targeting of proximal CA1 axons to the distal subiculum (Berns et al., 2018). Tenm3 also regulates the connectivity of orientation-selective RGCs in the zebrafish retina and optic tectum (Antinucci et al., 2013, 2016).

Thus, homophilic and heterophilic *trans*-interactions between specific CAMs generate a combinatorial recognition code for synaptic matching in addition to signaling for axon growth termination and synapse formation. This code not only specifies connectivity between distinct sets of neurons but can also direct synaptic specificity at a sub-cellular level through the accumulation of CAMs at defined sites. In the cerebellum, for instance, the IgSF CAM Neurofascin-185 accumulates in the axon initial segment (AIS) of Purkinje cells and directs the formation of pinceau synapses by basket interneurons (Ango et al., 2004). Similarly in the cortex, anchoring of L1CAM to the AIS of pyramidal neurons is required for selective innervation by Chandelier cells (Tai et al., 2019). The code specifying cellular or sub-cellular synaptic interactions is most often generated by the differential expression of CAMs among neurons but can also be achieved by the temporal regulation of CAM expression. In Drosophila, for instance, N-Cadherin is expressed by both R7 and R8 photoreceptors, albeit at different levels and moments. N-Cadherin expression peaks in R8 cells at the time R8 axons arrive at their target layer in the medulla, leading to axonal innervation of that layer. In contrast, R7 axons that express high levels of N-cadherin at a later timepoint bypass the R8 target and terminate in a more distant layer (Petrovic and Hummel, 2008). Alternatively, CAM *trans*-interactions can be modulated by alternative splicing. Splicing of Tenm2, for instance, changes the structure of Tenm2’s extracellular β-propeller domain and determines which *trans*-synaptic partner Tenm2 interacts with (Li et al., 2018, 2020). Tenm2 lacking the splice insert interacts with Latrophilins to promote excitatory synapse formation, whereas Tenm2 containing the insert cannot and specifies inhibitory synapses instead. Contact-mediated signaling between matching *trans*-synaptic partners eventually initiates synapse formation and stabilization by recruiting additional CAMs such as neurexins, neuroligins and LRR-containing adhesion molecules. As for synaptic matching, the assembly of synapses with specific properties is governed by the type of *trans*-synaptic molecules engaged. This vast field of research falls outside the scope of this review, but we refer interested readers to excellent recent articles on that topic (Schroeder and de Wit, 2018; Gomez et al., 2021; Südhof, 2021).

## Beyond Glue: The Non-Adhesive Functions of CAMs in Circuit Wiring

Although the adhesive properties of CAMs play critical roles at all stages of circuit wiring, CAM functions go far beyond adhesion and mechanical stabilization. Through their intracellular domains and interactions with other receptors at the plasma membrane, CAMs can activate or modulate a panoply of intracellular signaling pathways leading to morphological or transcriptional changes. They can also be cleaved from the plasma membrane and act as bona fide signaling ligands in the extracellular environment. CAMs have thus emerged as major signaling orchestrators of nervous system assembly.

### Transcriptional Regulation of Axon Growth

Axons elongating over long distances require the synthesis of new raw materials to sustain the assembly of cytoskeletal structures and membrane components. While local mRNA translation can be activated in growth cones to induce rapid changes in response to local cues (Dalla Costa et al., 2020; Agrawal and Welshhans, 2021), a constant communication between the growth cone and the nucleus regulates nuclear transcription to adjust gene expression to axonal needs. An increasing body of evidence indicates that CAMs regulate transcription in developing neurons, both indirectly by regulating the activation or trafficking of transcription factors, and directly, by acting in the nucleus themselves.

**FIGURE 3:**
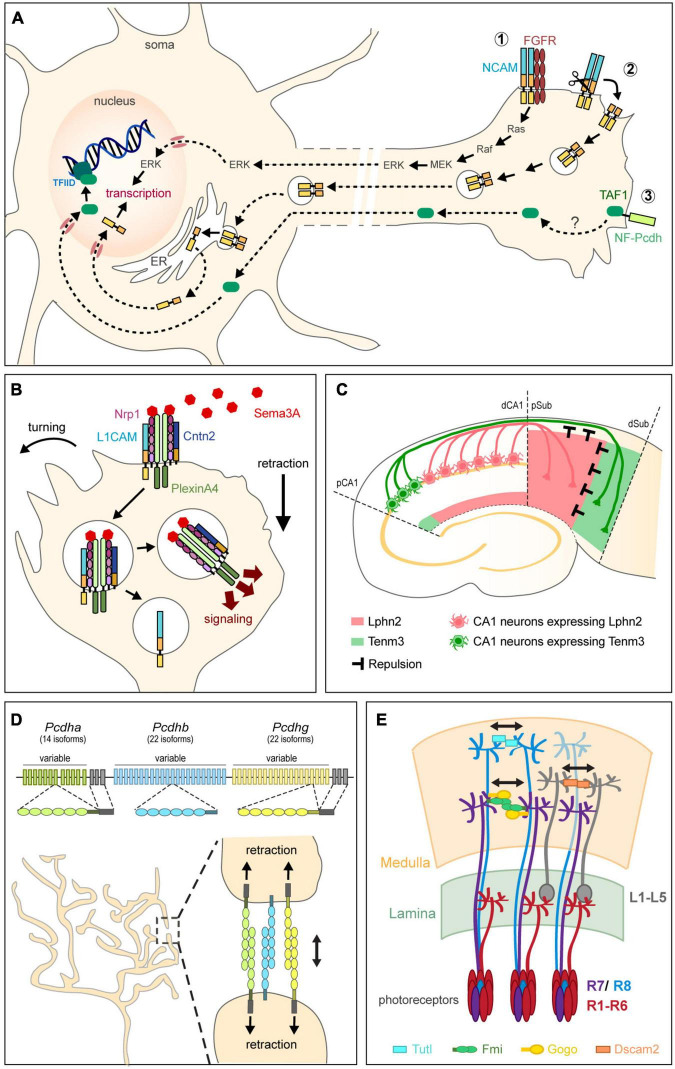
Non-adhesive functions of CAMs in circuit assembly. **(A)** CAMs regulate transcription for axon growth by activating intracellular signaling pathways from the plasma membrane (1), acting directly in the nucleus after proteolytic cleavage (2), or regulating the transport of molecules with transcriptional activity (3). NCAM, for instance, interacts with FGFR to activate the MAPK pathway and in turn, transcription (1). Alternatively, proteolytic processing of NCAM releases a fragment that is trafficked through endosomes and the endoplasmic reticulum (ER), released in the cytoplasm, and finally translocated into the nucleus where it regulates transcription (2). NF-protocadherin (NF-Pcdh) directly interacts with TAF1, a component of the basal transcription factor complex TFIID, suggesting that NF-Pcdh might regulate axon elongation through TAF1-mediated transcriptional control. **(B)** CAMs instruct axon repulsion by modulating signaling pathways. Both L1CAM and Cntn2 form a complex with Nrp1, the receptor to the repulsive guidance cue Sema3A, at the plasma membrane. Cntn2 modulates axon response to Sema3A by regulating the endocytosis of the Nrp1/L1CAM/Sema3A complex. After internalization, L1CAM and Nrp1 become segregated by Cntn2 into two distinct trafficking pathways. Nrp1 is routed to endocytic compartments where its increased association with PlexinA4 signals for collapse. Adapted from Law et al. (2008) and Dang et al. (2012). **(C)** CAMs instruct repulsion by acting as guidance cues. In the hippocampus, reciprocal repulsions mediated by Tenm3 and Lphn2 ensure proper target selection. Axons originating from proximal CA1 (pCA1) neurons (green) express Tenm3 and project to the distal subiculum (dSub) after being repelled by Lphn2 (pink) present in the proximal subiculum (pSub). Conversely, distal CA1 (dCA1) axons expressing Lphn2 (pink) are repelled by Tenm3 in dSub (green) and project to pSub. Adapted from Pederick et al. (2021). **(D)** Clustered Pcdhs regulate self-avoidance. Pcdh genes are organized into three adjacent clusters that include several variable exons. Each variable exon codes for an extracellular and transmembrane domains and is preceded by a promoter randomly activated in individual neurons to drive transcription. Stochastic promoter choice leads to the production of different Pcdh isoforms from each of the three clusters in a cell-specific manner, thereby generating a unique combination of Pcdh α, β, and γ expression in each neuron. Sister branches from the same terminal arbor express the same code of Pcdhs at their surface and repel each other after Pcdhs interact homophilically in *trans*. **(E)** CAMs ensure tiling of terminal arbors. In the Drosophila visual system, DSCAM2, Turtle (Tutl), and Flamingo (Fmi) together with Gogo engage in homophilic *trans*-interactions to activate repulsion, thereby control the proper spacing of L1-L5, R8, and R7 terminal arbors, respectively, in the medulla. Adapted from Spead and Poulain (2021).

Several CAMs stimulate gene transcription by activating intracellular signaling pathways, either through the activity of their intracellular domain or by interacting with signaling receptors (Figure 3A). L1CAM and NCAM, for instance, were both reported to activate mitogen-activated protein kinase (MAPK) pathways to promote neurite outgrowth (Kolkova et al., 2000; Schmid et al., 2000; Poplawski et al., 2012). NCAM interacts with the fibroblast growth factor receptor (FGFR) to activate MAPK and, in turn, the transcription factors CREB and c-Fos (Jessen et al., 2001; Niethammer et al., 2002). Activation of MAPK by L1CAM, on the other hand, requires L1CAM internalization (Schaefer et al., 1999; Schmid et al., 2000). Alternatively, CAMs might regulate transcription by directly modulating the nuclear trafficking of proteins with transcriptional activity. The intracellular domain of NF-protocadherin, for instance, directly binds TAF1, a component of the basal transcription factor complex TFIID (Heggem and Bradley, 2003). Inhibiting either NF-protocadherin or TAF1 severely impairs retinal axon initiation and elongation, suggesting that NF-protocadherin might regulate TAF1-dependent transcriptional programs to promote axonal growth (Piper et al., 2008). Conversely, classical cadherins constitutively retain the transcriptional coactivators β-catenin and p120 at the plasma membrane, thereby preventing them from translocating to the nucleus (Nelson and Nusse, 2004). Whether such a sequestration contributes to the transcriptional regulation of axonal development remains, however, unclear.

In addition to activating transcriptional pathways from the plasma membrane, a number of CAMs have recently emerged as transcriptional activators or repressors acting directly in the nucleus for controlling axon elongation (Figure 3A). Indeed, several proteases including caspases, matrix metalloproteases, and members of the ADAM family, can cleave the intracellular domains of transmembrane CAMs, which then translocate to the nucleus to regulate transcription. Activation of NCAM, for instance, leads to its cleavage by a serine protease and the subsequent nuclear import of a C-terminal fragment that is necessary for NCAM-induced axon growth (Kleene et al., 2010; Homrich et al., 2018). Interestingly, the fragment generated after cleavage includes not only the intracellular domain of NCAM but also its transmembrane domain and a stub of its extracellular domain, indicating that NCAM is proteolytic processed by an extracellular enzyme. Modification of the extracellular stub by polysialic acid, a glycan known to modulate NCAM function, does not prevent its nuclear import but leads to the transcription of a distinct set of genes, demonstrating a unique role for NCAM glycosylation in transcriptional regulation (Westphal et al., 2016, 2017a,b). L1CAM, DSCAM and DSCAML1 also regulate transcription after cleavage. Like NCAM, activated L1CAM undergoes a serine protease-dependent cleavage at the plasma membrane that generates a fragment containing the intracellular, transmembrane and part of the extracellular domains (Lutz et al., 2012, 2014). The sumoylated L1CAM fragment hence generated traffics to endosomes and the cytoplasm before translocating in a sumoylation-dependent manner to the nucleus where it interacts with multiple nuclear proteins (Girbes Minguez et al., 2020). Interaction with heterochromatin protein 1 is notably required for L1CAM-mediated neurite outgrowth in cultured cortical neurons (Kleene et al., 2022). Conversely, the intracellular domains generated after cleavage of DSCAM and DSCAML1 by γ-secretase alter the transcription of genes regulating circuit formation and inhibit axon growth when overexpressed in cortical neurons (Sachse et al., 2019). Other CAMs besides IgSF CAMs appear to directly regulate transcription in the nucleus. The intracellular domain of Tenm2, for instance, is released after homophilic interaction and represses the activity of Zic1, a transcription factor known to regulate the targeting of mossy fibers in the cerebellum (Bagutti et al., 2003; Dipietrantonio and Dymecki, 2009). Conversely, Tenm3’s intracellular domain interacts with Zic2, another member of the Zic family that specifies binocular vision by regulating the guidance of retinal axons projecting ipsilaterally in the visual system (Herrera et al., 2003; Glendining et al., 2017). Both Zic2 and its transcriptional target EphB1 are upregulated in Tenm3 mutants, which likely explains the strong ipsilateral targeting defects observed in these mice (Leamey et al., 2007; Dharmaratne et al., 2012). As for Tenm1, its intracellular domain has been shown to regulate transcription by binding to the transcriptional repressors MBD1 and HINT1 (Nunes et al., 2005; Schöler et al., 2015). Defining the mechanisms controlling the release and transport of CAM intracellular domains, and identifying the cell-specific nuclear partners they interact with, remain important questions to address for better understanding how long distance communication between axons and soma modulate axon guidance at choice points and target innervation.

### Repulsive Signaling for Axon Pathfinding

Although CAMs were first shown to promote axon growth through their adhesive properties, many of them have since emerged as signaling receptors or ligands instructing growth cone guidance independently of adhesion. Perhaps unexpectedly, CAMs were found to actively participate in the control of axon repulsion by dictating the axon’s sensitivity to repulsive signals. Many IgSF CAMs, for instance, form signaling complexes with receptors to repulsive guidance cues. L1CAM directly binds in *cis* to Neuropilin-1 (Nrp1), the receptor to Sema3A, and is required for Sema3A-induced growth cone collapse (Castellani et al., 2000, 2002). L1CAM mediates both signaling downstream of Nrp1 and Nrp1 internalization upon Sema3A binding, thus coordinating signaling cascades instructing growth cone collapse with a decreased adhesiveness (Castellani et al., 2004; Bechara et al., 2008). Interestingly, Cntn2 also forms a complex with Nrp1 and L1CAM at the plasma membrane and regulates axon response to Sema3A by modulating the endocytosis of the Nrp1/L1CAM/Sema3A complex (Law et al., 2008; Figure 3B). L1CAM and Nrp1 are endocytosed together but become segregated by Cntn2 into two distinct trafficking pathways (Dang et al., 2012). Nrp1 is notably routed to endocytic compartments where it increases its association with PlexinA4, which in turn signals for collapse. In the absence of Cntn2, Nrp1, and L1CAM are no longer separated intracellularly and signaling is reduced. Similarly to L1CAM that associates with Nrp1, NrCAM forms a complex with Nrp2 and PlexinA3 (Falk et al., 2005; Demyanenko et al., 2011). In response to Sema3F, NrCAM clusters Nrp2 and PlexinA3 at the plasma membrane, which activates signaling for growth cone collapse. Thalamocortical axons lacking NrCAM are no longer sensitive to Sema3F *in vivo* and misproject caudally in the ventral telencephalon (Demyanenko et al., 2011). Other guidance pathways besides Semaphorin signaling are controlled by IgSF CAMs. NCAM, for instance, clusters and activates EphA3 in response to ephrin-A5, thereby eliciting RhoA-dependent growth cone collapse in GABAergic interneurons (Sullivan et al., 2016). Similarly, DSCAM interacts with Unc5 to trigger the repulsion of cerebellar axons away from Netrin-1 (Purohit et al., 2012). More recently, the LRR-containing adhesion molecule FLRT3 was shown to directly interact with Robo1, a receptor to Slits (Leyva-Díaz et al., 2014). FLRT3 not only modulates the repulsion of rostral thalamocortical axons in response to Slit1 *in vitro*, but also their attraction toward Netrin-1 in a Robo1-dependent manner *in vivo*.

In addition to forming signaling complexes with guidance receptors, CAMs can act directly as instructive cues for axon repulsion. In Drosophila, for instance, Integrins α1 and α2 mediate *trans-*axonal repulsive signaling between motor axons to induce their defasciculation and target them to proper targets (Huang et al., 2007). Mutants lacking either of these integrins have increased axon fasciculation that causes a lack of muscle innervation. Likewise *in vitro*, the close homolog of L1CAM, Chl1, engages in homophilic interactions to repel ventral midbrain dopaminergic axons (Alsanie et al., 2017). Very recently, repulsive interactions between Tenm3 and Lphn2 were shown to topographically direct CA1 axons to the subiculum in the hippocampus (Pederick et al., 2021; Figure 3C). CAMs can instruct axon repulsion not only locally by signaling from the surface of cells or others axons, but also distally by acting as a gradient after shedding of their extracellular domain. The ectodomains of FLRT2 and FLRT3, for instance, are released after cleavage by metalloproteases and act as repulsive guidance cues for hippocampal axons expressing Unc5 (Yamagishi et al., 2011). While many CAMs are processed by metalloproteases (Saftig and Lichtenthaler, 2015), the functions of their ectodomains in circuit assembly remain surprisingly unknown. We can anticipate, though, that ectodomain release would increase the functional diversity of CAMs and provide an additional level of spatiotemporal regulation for signaling. The ectodomain of Tenm2, for instance, is proteolytically cleaved during development and was recently shown to attract hippocampal axons *in vitro* by binding to Lphn1 (Vysokov et al., 2016, 2018).

### Contact-Mediated Self-Avoidance and Tiling

Neuronal connectivity and function rely on the precise innervation of targets by axons. After reaching their final destination, axons branch extensively to form elaborate terminal arbors within specific territories. Branching patterns form dynamically and become spatially organized to maximize the coverage of an area while minimizing redundancy of targeting. Optimal coverage is achieved through two main mechanisms (Grueber and Sagasti, 2010). Isoneural spacing or self-avoidance refers to the repulsion between axonal branches of a same neuron, so that branches avoid overlapping with each other. Likewise, arbors from distinct neurons that share the same function do not overlap, a phenomenon referred to as heteroneural avoidance or tiling. Both self-avoidance and tiling pertain to axons as well as dendrites and are achieved by contact-mediated repulsion (Sagasti et al., 2005).

First described in studies analyzing the receptor fields of sensory neurons in the leech (Nicholls and Baylor, 1968), self-avoidance relies on the ability of sister branches from the same terminal arbor to discriminate “self” from “non-self” before repelling each other. This selective recognition is achieved by the expression of a cell-surface molecular code that is common to sister branches but distinct among branches from different neurons. In Drosophila, DSCAM1 generates such a code. Alternative splicing of DSCAM1 pre-mRNA produces 38,016 distinct isoforms that differ in their ectodomain and are expressed in a probabilistic way (Schmucker et al., 2000). Consequently, each isoform gives an individual neuron a unique molecular identity. As only identical ectodomains can engage in homophilic *trans-*interactions due to conformational constraints, only sister branches sharing the same isoform will recognize each other (Wojtowicz et al., 2004, 2007). DSCAM1-mediated homophilic interactions initiate repulsion rather than adhesion in this context, which contrasts with the known function of DSCAM in promoting axon fasciculation (Bruce et al., 2017). DSCAM1-mediated self-avoidance enables the proper spatial organization of both axonal and dendritic arbors in diverse neuronal populations (Wang J. et al., 2002; Zhan et al., 2004; Hattori et al., 2007; Matthews et al., 2007; Soba et al., 2007). In the absence of DSCAM1, branches fail to separate and overlap or fasciculate instead. Interestingly, although the vertebrate ortholog DSCAM and its homolog DSCAML mediate self-avoidance in the mouse retina (Fuerst et al., 2008, 2009), they are not highly spliced and thus do not generate a molecular code like DSCAM1. Instead, DSCAM appears to mask adhesion-promoting signaling from other CAMs like cadherins (Garrett et al., 2018). If not DSCAM, which cell-surface molecules generate a recognition code in vertebrates? Like DSCAM1, clustered Protocadherins (Pcdhs) exist in a multitude of isoforms and as such, represent the largest subgroup in the Cadherin superfamily. About 60 Pcdh genes are organized into three adjacent clusters designed as Pcdh α, β, and γ (Wu and Maniatis, 1999; Figure 3D). Each cluster includes several variable exons that each codes for an extracellular and transmembrane domains. Each variable exon is further preceded by a promoter that is randomly activated in individual neurons to drive transcription (Tasic et al., 2002; Wang X. et al., 2002). Thus, stochastic promoter choice leads to the production of different Pcdh isoforms from each of the three clusters in a cell-specific manner, thereby generating a unique combination of Pcdh α, β, and γ expression in each neuron (Esumi et al., 2005; Kaneko et al., 2006; Thu et al., 2014; Mountoufaris et al., 2017). Clusters α and γ further include constant exons coding for a common intracellular domain that are combined to each variable exon by alternative splicing. Like DSCAM1, Pcdhs engage in strict homophilic *trans-*interactions and as such, provide a recognition code for sister branches of the same neuron (Hasegawa et al., 2012; Thu et al., 2014; Mountoufaris et al., 2017). They also form multimers in *cis* at the plasma membrane and signal through the intracellular domain of Pcdhs α and γ to initiate repulsion. Thanks to their diversity, Pcdhs have emerged as the main mediators of axonal and dendritic self-avoidance in vertebrates (Lefebvre et al., 2012). Deleting all three Pcdh clusters in mice, for instance, causes a loss of self-avoidance and severe arborization defects in olfactory axons (Mountoufaris et al., 2017). Conversely, “erasing” the Pcdh code by overexpressing a single-tricluster gene repertoire prevents olfactory axons from converging into stereotypically positioned glomeruli in the olfactory bulb.

Protocadherins also appear to regulate axonal tiling in addition to self-avoidance. In the basal ganglia and hippocampus, for instance, serotonergic axon terminals are evenly spaced in their target fields but become clumped together in mice lacking Pcdhαc2, the only Pcdhα isoform expressed in serotonergic neurons (Chen et al., 2017). Whether tiling of other axonal populations is similarly regulated by the expression of unique Pcdhs remains to be determined. Other CAMs, however, have been identified as tiling regulators. DSCAM is notably required for proper axonal and dendritic tiling of bipolar cells in the retina (Simmons et al., 2017). Likewise in the Drosophila visual system, DSCAM2, the atypical cadherin Flamingo (Fmi), and the IgSF CAM Turtle (Tutl) enable proper spacing of distinct classes of axons in spatially restricted columns in the medulla (Millard et al., 2007; Tomasi et al., 2008; Ferguson et al., 2009; Hakeda-Suzuki et al., 2011; Figure 3E). Tutl engages in homophilic *trans*-interactions to prevent adjacent R7 photoreceptor cell terminals from overlapping (Ferguson et al., 2009). Similarly, Fmi mediates repulsive *trans*-interactions between R8 photoreceptors axons. Fmi interacts in *cis* with Gogo, another transmembrane receptor eliciting repulsive axon-axon interactions, suggesting that both proteins might act as a complex at the surface of branches to ensure tiling (Tomasi et al., 2008; Hakeda-Suzuki et al., 2011). Lastly, DSCAM2 ensures proper spacing of L1 axonal arbors, thereby restricting them to specific columns. In *dscam2* mutants, L1 axons still innervate the correct layer of the medulla but are no longer restricted to a single column (Millard et al., 2007). Altogether, these studies indicate that the selective expression of individual or combined CAMs generates a cell-surface recognition code that ensures the segregation of terminal arbors into non-overlapping domains while instructing laminar targeting and synaptic specificity.

## Conclusion and Perspectives

In conclusion, a large body of literature now demonstrates that CAMs orchestrate a striking number of developmental processes that are critical for neural circuit wiring. By engaging in homophilic and heterophilic *trans*-interactions, CAMs not only provide the adhesion and mechanical stabilization required for proper axon guidance and fasciculation, but also generate a cell-surface recognition code essential for synaptic specificity and contact-mediated self-avoidance and tiling. CAMs further act as bona fide signaling factors instructing growth cone behavior, modulating the activities of guidance receptors, and controlling transcriptional programs for axonal development.

Surprisingly, whether CAMs also contribute to the regressive events that refine neural circuits remains poorly known. Selective axon degeneration, for instance, is used to remodel axonal projections during metamorphosis in insects or prune exuberant axons or axonal branches in vertebrates (Riccomagno and Kolodkin, 2015; Schuldiner and Yaron, 2015). In the visual system, retinal axons that missort along the optic tract selectively degenerate, leading to proper pre-target topographic ordering of retinal projections (Poulain and Chien, 2013). Refinement of terminal retinal arbors also occurs at the target in an activity-dependent manner, leading to the sharpening of retinotopic maps and the segregation of ipsi- and contralateral axons into eye-specific territories in animals with binocular vision (Stellwagen and Shatz, 2002; Chandrasekaran et al., 2005; Ben Fredj et al., 2010). Interestingly, Cntn2 was recently identified as a key regulator of retinotectal map sharpening in zebrafish (Spead et al., 2021). Nasal retinal axons progressively refine their projection domain to the posterior tectum in wild-type but not in the absence of Cntn2, causing a lack of retinotopic map refinement along the antero-posterior axis. How Cntn2 remodels axon terminal arbors remains to be determined but might involve an interplay between Cntn2 signaling and neuronal activity. Indeed, neuronal activity might modulate the targeting of Cntn2 to the plasma membrane, just as it regulates that of Cntn1 in hypothalamic axons (Pierre et al., 2001). Conversely, Cntn2 could modulate neuronal activity by regulating sodium or potassium channels. Cntn1, for instance, interacts with several voltage-gated sodium channels and increases their density at the plasma membrane (Kazarinova-Noyes et al., 2001; Liu et al., 2001). Dissecting the cross-talk between CAMs and other signaling pathways will be critical to fully comprehend the multiple roles CAMs play at various stages of circuit assembly. It might also provide new strategies for correcting mechanisms that get dysregulated in the context of neurodevelopmental disorders.

## Author Contributions

Both authors listed have made a substantial, direct, and intellectual contribution to the work, and approved it for publication.

## Conflict of Interest

The authors declare that the research was conducted in the absence of any commercial or financial relationships that could be construed as a potential conflict of interest.

## Publisher’s Note

All claims expressed in this article are solely those of the authors and do not necessarily represent those of their affiliated organizations, or those of the publisher, the editors and the reviewers. Any product that may be evaluated in this article, or claim that may be made by its manufacturer, is not guaranteed or endorsed by the publisher.
